# Wessex Branch of the Association of Clinical Pathologists, Autumn Meeting 1989

**Published:** 1990-09

**Authors:** 


### Wessex Branch Association of Clinical Pathologists Autumn Meeting

### Meeting held at Postgraduate Medical Centre, Musgrove Park Hospital, Taunton, Saturday 11 November, 1989

**The winter meeting of the Wessex Branch of the Association of Clinical Pathologist was held at the Postgraduate Centre, Musgrove Park Hospital, Taunton on Saturday 11 November 1989.**

**Professor Peter Anthony, President of the Branch, took the chair for the business meeting. For the scientific meeting, which by tradition consists mainly of papers by junior members, the chair was taken by Dr A E Adams (Taunton) in the morning and Dr S C Smith (Taunton) in the afternoon. Nearly 40 pathologists attended.**

### MUCOSAL PROLAPSE — A POSSIBLE ORIGIN FOR METAPLASTIC POLYPS

**B F Warren, J D Davies**
Bristol Royal Infirmary, Bristol

The cause of metaplastic colonic polyps is unknown. In the past, their relationship to adenomas has been debated repeatedly. Cases of prolapsing rectal mucosa syndrome frequently show surface epithelial metaplasia. Examination of the base of metaplstic polyps often reveals muscular disruption. The mucin histochemistry of metaplastic polyps and prolapsing rectal mucosa syndrome is similar. We have described previously the occurrence of diamond shaped crypts and intramucosal elastic fibres in prolapsing rectal mucosa syndrome. The histological distinction between larger metaplastic polyps and smaller polypoid manifestations of the prolapsing rectal mucosa syndrome is often extremely difficult.

Twenty-five rectal metaplastic polyps of varying size were examined by light microscopy for the presence of muscular disruption, diamond shaped crypts, fibroplasia of the lamina propria, and intramucosal elastic fibres. The largest polyp examined was 5 mm. Nineteen polyps showed muscular disruption, 14 had muscle fibres within the lamina propria, 14 contained diamond shaped crypts, 14 fibroplasia of the lamina propria, and 18 had intramucosal elastic fibres. In view of these shared histological features, we suggest that rectal metaplastic polyps may be extremely localised forms of mucosal prolapse.

### THE EFFECT OF VITAMIN E SUPPLEMENTATION UPON SOME INDICES OF FREE RADICAL ACTIVITY IN INSULIN-DEPENDENT DIABETES

**A A McConnell**
Southmead Hospital, Bristol

Excess free radical activity is thought to occur in diabetes and may contribute to the development of diagetic complications. This study was carried out to investigate the generation of free radicals, in particular the superoxide radical anion, and also to assess the defence against free radicals in the form of serum vitamin A and E concentrations and caeruloplasmin concentrations, both in a group of insulin-dependent diabetics (number 17) and in an age-matched group of normal control subjects (number 15).

Neutrophil suspensions were used to study the generations of superoxide radical anion, both in the resting state and following stimulation with phorbol myristate acetate, PMA. Superoxide dismutase inhibited these reactions, confirming their dependence on superoxide radical anion. The resting rate of radical production was similar in the diabetic and the control groups, but the production rate in response to PMA was significantly increased in the diabetic group. Supplementation with vitamin E had little effect on these measurements.

Baseline measurements revealed that the diabetic group had higher levels of vitamin E and of caeruloplasmin than the control group. Supplementation produced an increase in vitamin E levels, but a fall in caeruloplasmin levels in both groups. Baseline vitamin A levels were similar in the two groups but fell significantly only in the diabetic group.

### A NEW GASTRIC SPIRAL BACTERIUM

***Gastrospirillum hominis, gen. nov., sp. nov.***

**Cliodna A M McNulty, Julie C Dent, A Curry*, J C Uff, G A Ford, S P Wilkinson, M W L Gear**
Gloucester Public Health Laboratory and *Public Health Laboratory, Manchester

We describe a new spiral bacterium, distinct from *C. Pylori*, which we found in the gastric mucosa of 6 patients with gastrointestinal symptoms. The organisms were not merely contaminants from the mouth as they were found more than once in the same patients and were found beneath the mucus and within the necks of the pyloric glands. All patients had chronic active type B gastritis and four had oesophagitis. All the patients had another organic cause for their gastrointestinal symptoms. The organism is helical, 3–7μm long and 0.9 μm in diameter with truncated ends flattened at the tips. The oganism has an electron dense thickening on the protoplasmic surface of the terminal region and up to 12 sheathed flagella 28 nm in diameter at each pole. The new spiral bacterium doies not conform to any known genus but might reasonably be placed in the family *Spirillacae*. We propose a new genus "*Gastrospirillum*" (Gr. n. *gastr*, pertaining to the stomach; Gr. n. *spira* a spiral; M.L. dim. neut. N. *spirillum* a small spiral) which should include all the spiral bacterium other than *C. pylori* found in the gastric mucosa of animals. We suggest that the human spiral bacterium should be called "*Gastrospirillum hominis*".

### THE FARNSWORTH–MUNSELL TEST — ONE IN THE EYE FOR HISTOPATHOLOGY

**H S Rigby**
Bristol Royal Infirmary, Bristol

Tinctorial stains such as those used in the study of colonic mucins can be expensive, technically difficult, and time consuming. Their interpretation depends upon the histopathologist's ability to differentiate between hues. It has been assumed, without formal testing, that pathologists are able colour discriminators.

The colour discrimination ability of 30 pathologists of varying experience was assessed using Farnsworth–Munsell 100 Hue test.

Twenty-eight showed a wide ranging ability to differentiate colours, but none were colour blind. As a group, they performed better than a randomly selected population sample. However, 10% of the sample still fell below the twentieth centile for normals. These individuals may, unknowingly, misinterpret stains. Individuals with the least aptitude for colour discrimination were not those with the least pathological experience.

Two individuals tested had specific and major defects which could possibly affect their ability in the interpretation of a wider range of less subtle stains.

Pathologists should, therefore, be made aware of their colour discrimination ability, and perhaps formal colour vision testing of prospective histopathologists is appropriate.

### LYME ARTHRITIS IN CHILDHOOD

**J Doris**
Bristol

Lyme disease is now clearly recognised to result from infection with the tick-borne spirochaete, *Borrelia burgdorferi*. Like other spirochaetal infections, the clinical manifestations of Lyme disease are variable. Classically, it is associated with a rash, erythema chronicum migrans which occurs at the site of the tick bite. Other manifestations include cranial nerve palsy, cardiac conduction defects and arthritis. Indeed, it was the close geographic clustering of cases in the community of Lyme, Connecticut, USA, which first aroused suspicion that an infective agent was responsible for an outbreak of arthritis in children in the mid-seventies.

In the United States, Lyme arthritis is seen in both adults and children. However, arthritis as a complication of infection seems far less common in Europe. This may result from subtle antigenic differences between American and European borreliae. We describe a case of childhood arthritis. To our knowledge, no case of childhood arthritis caused by Lyme infection has previously been described in the UK. It is important that such cases be identified so that appropriate antibiotic therapy with penicillin or tetracycline be instituted. Untreated, the disease can progress to an erosive arthropathy.

### CYTOLOGY AS A DIAGNOSTIC TOOL IN THE DIAGNOSIS OF BLADDER TUMOUR; THE EFFECT OF CELL PREPARATION

**Jennifer Dhundee**
Southmead Hospital, Bristol

The aim of the study was to compare the ability of urine cytology to detect urinary tract malignancies with two techniques of smear preparation. Using computer based files, the reports of urine cytology requests for two six-month periods were compared.

From July to December 1987 urine samples were fixed after the smear was made (method 1) and from July to December 1988 the cells were fixed singly using Esposti's fluid prior to smear preparation (method 2). The cytological diagnoses were correlated with the final histological diagnoses. The specificity of urine cytology to detect malignancy for both methods was high (99%). However, the sensitivity using method 1 was 87% whereas that using method 2 was 65%. This difference was statistically significant.

It was concluded that cell preparation techniques affect the usefulness of urinary cytology and that cellular fixation following smear preparation is preferable to fixation prior to smear preparation.

### IS THE ENDOMETRIUM A "STERILE" ENVIRONMENT?

**P Cowling, D R McCoy and R Marshall**
Southmead Hospital, Bristol

Following work in the USA claiming successful treatment of pre-menstrual tension with doxycycline which was the subject of a "Sunday Times" article, there were a number of women requesting antibiotics for this condition. Our study was designed to show that the endometrium is microbiologically sterile.

One hundred uteri removed abdominally from premenopausal women were opened with a sterile scalpel and the endometrium curetted. The curettings were placed in a maintenance broth and homogenised before inoculating onto NY City Agar, Blood Agar, Fastidious Anaerobe Agar, Chocolate Agar, and an "Imagen" slide (for chlamydiae). The results were as follows:

| *Organism* | *No. Isolates** | *Organism* | *No. Isolates* |
|---|---|---|---|
| Lactobacillus | 11 (1) | Strep. faecalis | 1 |
| Mycoplasma hominis | 6 (3) | Strep. milleri | 1 (1) |
| Staph. epidermidis | 3 (1) | E. coli | 1 |
| Anaerobic cocci | 3 (2) | C. jeikieum | 1 |
| Kingella kingii | 2 | Ureaplasma sp. | 1 (1) |
| Moraxella urethralis | 1 | Chlamydiae | 0 |
| Strep. agalactiae | 1 | No growth | 71 |
| Strep. viridans | 1 (1) | | |

* Figures in parentheses = No. isolates growing in the presence of one other organism.

Twenty-two percent of endometria gave positive cultures in our study. This is in contrast to some previous studies in which much higher percentages are quoted. Our method avoids contamination by cervical flora which may explain the lower isolation rate.

In conclusion, the endometrium is not always sterile, a wide variety of organisms may colonize the site. Work is continuing in an attempt to define factors involved in the colonization process.

### HIGH GRADE B-CELL LYMPYHOMA ASSOCIATED WITH DIFFUSE ERYTHRODERMA

**B H Ramsahoye**
Bristol

This presentation of non-Hodgkin's lymphoma with localised or diffuse skin infiltration is normally considered a phenomenon of T-cell lymphoma (Mycosis fungoides or Sezary syndrome). The infiltrate may be manifested by localised patches, plaques or a diffuse erythemtous rash. Localised skin infiltration by B-cell lymphoma has been described, but usually as a late manifestation of pre-existing disease. B-cell lymphoma presenting as a diffuse skin rash has not, to our knowledge, been described.

We present a 79 year old man who presented five months prior to his first manifestation of centroblastic B-cell lymphoma (testicular disease) with a diffuse erythematous skin rash with areas of dermal thickening and induration. Treatment with topical steroids caused no improvement but within three days of systemic chemotherapy total resolution of the rash had occurred.

Skin biopsy was undertaken 24 hours post chemotherapy. Histology showed infiltrtion consistent with lymphoma but marker studies failed to show B-cell clonality. Definitive B-cell infiltration of the skin could therefore not be proved. The temporal association of a diffuse rash occurring for the first time in a 79 year old man with established lymphoma and complete resolution with chemotherapy tends to indicate an association between the two conditions. We postulate that presentation of high grade B-cell lymphoma with diffuse dermal dinfiltration does occur, but definitive proof is still required.

### A CASE OF AMEGAKARYOCYTIC THROMBOCYTOPENIA RESPONSIVE TO STEROID THERAPY

**L Jones**
Southmead Hospital, Bristol

Acquired thrombocytopenia due to aplasia of megakaryocytes without other haematopoietic abnormalities or relation to drugs is a rare condition. To our knowledge, only ten adult cases have been reported. The possible pathogenic mechanisms appear to be an intrinsic defect of megakaryocytic colony-forming units (M-CFUs). However, in some patients, a circulating cytotoxic autoantibody to M-CFUs has been described. Death due to thrombocytopenia or progression to aplastic anaemia or amyelodysplastic syndrome is the rule.

We present a patient with acquired amegakaryocytic thrombocytopenia with low T8 (cytotoxic/suppression) cell numbers and a 30% infiltration by polyclonal lymphocytes. Response to antilymphocyte globulin and steroids (twice) and steroids alone (once) was seen. Relapse occurred after steroid withdrawal. This is the first recorded response to immunosuppression by steroids in amegakaryocytic thrombocytopenia. We postulate that immune mechanisms are occasionally responsible for this condition.

